# Risk‐Based Triage Strategy by Extended HPV Genotyping for Women With LSIL Cytology: A Real‐World Study

**DOI:** 10.1002/jmv.70404

**Published:** 2025-05-20

**Authors:** Chun Ye, Yi Liu, Huiru Huang, Ruizhe Chen, Ying Li, Xiaofei Zhang, Yunfeng Fu, Liang Feng, Xiao Li

**Affiliations:** ^1^ Department of Gynecologic Oncology, Women's Hospital Zhejiang University School of Medicine Hangzhou China; ^2^ Medical Centre for Cervical Diseases, Women's Hospital Zhejiang University School of Medicine Hangzhou China; ^3^ Department of Gynecology and Obstetrics Xixi Hospital of Hangzhou Hangzhou China; ^4^ Department of Pathology, Women's Hospital Zhejiang University School of Medicine Hangzhou China; ^5^ Zhejiang Provincial Center for Disease Control Hangzhou China; ^6^ Zhejiang Provincial Key Laboratory of Precision Diagnosis and Therapy for Major Gynecological Diseases Hangzhou China; ^7^ Zhejiang Provincial Clinical Research Center for Gynecological Diseases Hangzhou China

**Keywords:** cervical intraepithelial neoplasia, extended HPV genotyping, human papillomavirus, low‐grade squamous intraepithelial lesion, risk‐based triage strategy

## Abstract

To evaluate the immediate risk of (pre)cancer for cytology low‐grade squamous intraepithelial lesion (LSIL) women infected with or without specific HPV genotype and develop a risk‐based management strategy. A total of 4567 LSIL women with extended HPV genotyping and colposcopy results were enrolled according to the inclusive and exclusive criteria. The distribution and immediate cervical intraepithelial neoplasia grade 2 or worse and 3+ or worse (CIN2+/3+) risks of specific HPV genotypes were assessed using Minimum Estimate, Any Type Estimate, and Hierarchical Attribution Estimate. A risk‐based strategy was further established and evaluated. CIN2+/3+ were 729/328 cases, including 691/317 in 3398 HPV‐positive and 38/11 in 1169 HPV‐negative women. HPV16, 52, 58, and 18 were the most prevalent genotypes in both HPV‐positive and CIN2+/3+ cases. HPV16, 73, and 33 carried the highest immediate CIN2+/3+ risk. A risk‐based strategy was established, which suggested Group A (HPV 16, 33, 45, 31, 18, 58, 52, 35, 73, 82; with immediate CIN3+ risk of 4.08%–22.12%) for immediate colposcopy, Group B (HPV 59, 66, 56, 53) for 6‐month follow‐up or p16/Ki‐67 dual stain or DNA methylation triage, while Group C (HPV 51, 68, 39, 26) for 1‐year HPV repeat testing. Compared with conventional strategy, this new strategy showed significantly higher specificity (CIN2+: 52.16% vs. 29.47%, *χ* ^2^ = 409.136, *p* < 0.001; CIN3+: 48.45% vs. 27.32%, *χ* ^2^ = 402.395, *p* < 0.001) but similar sensitivity, which could reduce immediate colposcopy referrals by 19.82%. A risk‐based triage strategy for LSIL women with extended HPV genotyping could effectively reduce unnecessary colposcopies and maintain high efficacy for CIN2+/3+ detection.

**Abbreviations**
AC: adenocarcinoma
AIS: adenocarcinoma in situ
ASCCP: American Society for Colposcopy and Cervical Pathology
Any.: any type estimate
CC: cervical cancer
CIN: cervical intraepithelial neoplasia
CIN2+: cervical intraepithelial neoplasia grade 2 or worse
CIN3+: cervical intraepithelial neoplasia grade 3 or worse
CIN2+/3+: cervical intraepithelial neoplasia grade 2 or worse and grade 3 or worse
HPV: human papillomavirus
HrHPV: high‐risk HPV
Hier.: hierarchical attribution estimate
LSIL: Low‐grade squamous intraepithelial lesion
Min.: minimum estimate
NPV: negative predictive value
PPV: positive predictive value
SCC: squamous cell carcinoma
SPSS: Statistical Package for the Social Sciences
VaIN: Vaginal Intraepithelial Neoplasia

## 1 Introduction

Cervical cancer (CC) is the fourth most common cancer in terms of both incidence and mortality in women, with around 660 000 new cases and 350 000 deaths worldwide reported in 2022 [1]. Persistent infection with high‐risk human papillomavirus (HPV) is a major risk factor for the development of cervical precancer lesions and cancer [2, 3]. More than 400 HPV genotypes have been identified, with about 40 types colonizing the genital tract [4]. Their carcinogenic potential and prevalence vary geographically [5, 6]. Currently, 14 genotypes (16, 18, 31, 33, 35, 39, 45, 51, 52, 56, 58, 59, 66, and 68) are classified as high‐risk HPV (hrHPV), and 4 genotypes (26, 53, 73, and 82) as intermediate‐risk HPV [7]. Although HPV16 and 18 are the most potent carcinogens for cervical cancer, other HPV genotypes do not carry equal risk [6]. Studies on specific HPV type risks for CIN2+ and CIN3+ remain limited, and the findings are inconsistent [8, 9, 10]. Recently, the U.S. Food and Drug Administration (FDA) approved two extended genotyping Kit for identifying groups of HPV types beyond 16 and 18 [11]. China's National Medical Products Administration (NMPA) also approved several kits for further identifying individual non‐16/18 HPV types [12]. These extended genotyping methods provided the potential for more accurate risk assessment and better treatment decisions in infected women [9, 13].

Low‐grade squamous intraepithelial lesion (LSIL), a cytological change typically caused by HPV infection, is classified according to the 2014 Bethesda System (TBS) [14]. Its cytological features are usually relatively characteristic, leading to consistent interpretation. The prevalence of hrHPV infection in LSIL patients is substantial, ranging from approximately 61%–83%, with geographic and age variations [15, 16, 17, 18, 19, 20]. Previous studies have shown that hrHPV positive LSIL patients have a significantly increased risk of progressing to CIN2+ (12%–19%) compared to hrHPV negative patients (2%–8%) [16, 19, 21, 22]. According to the 2019 American Society for Colposcopy and Cervical Pathology (ASCCP) guidelines, the immediate risk of CIN3+ in hrHPV positive LSIL is 4.3%, compared to 1.1% in hrHPV negative LSIL [23, 24]. Consequently, conventional strategy generally recommends that hrHPV positive women with LSIL should be referred for colposcopy for cervical disease [24]. While hrHPV negative LSIL women have a relatively low risk of disease progression and may be considered for close follow‐up.

As we know, CIN3+ risk in HPV‐positive LSIL is only slightly higher than the colposcopy referral threshold of 4%. Extended genotyping can facilitate the timely detection of CIN2+/3+ lesions and reduce unnecessary colposcopy referrals [25, 26]. However, limited data were available about the role of specific hrHPV and intermediate‐risk HPV in cytology LSIL [27]. Moreover, many studies analyzed the risk of CIN2+ or CIN3+ separately [13, 28, 29], failing to comprehensively assess the overall risk of progression to CIN2+/3+. Further investigations into extended HPV genotyping could be valuable in identifying Chinese women at an elevated risk of cervical (pre)cancer.

In this study, we aimed to identify the immediate risk of developing CIN2+/3+ associated with specific hrHPV and intermediate‐risk HPV genotypes in women with LSIL cytology and to develop a precise management strategy based on the 2019 ASCCP principle of “equal management for equal risk”. By leveraging real‐world clinical data, we sought to improve the diagnostic efficiency of CIN2+/3+ in the LSIL population and reduce unnecessary colposcopies.

## 2 Methods

### 2.1 Study Subjects and Design

In this real‐world study, clinical information was retrospectively retrieved for women with LSIL cytology referred for colposcopy at the Women's Hospital, Zhejiang University School of Medicine, China, between July 1, 2014, and December 31, 2021. Women with LSIL who met all the following criteria were included: (1) HPV‐negative or HPV‐positive with extended genotyping results, (2) colposcopy and biopsy (and/or endocervical curettage, ECC) were performed within 3 months of LSIL cytology. Patients were excluded if they had: (1) Unclassified/unknown HPV genotypes, (2) a History of cervical cancer or total hysterectomy, (3) pregnancy, abortion, or delivery within 6 weeks. This study protocol was approved by the ethics committee of our hospital (IRB‐20240280‐R).

### 2.2 Variables

The variables were collected, including age, specific HPV genotype results, and histopathology reports. Colposcopies were performed by experienced colposcopists based on colposcopic impression and cervical screening results, and targeted or random biopsies and ECC (if needed after evaluation) were taken. Histopathology reports were made by two pathologists according to the 2020 WHO Classification of Female Genital Tumors, including normal, CIN1, CIN2, CIN3, adenocarcinoma in situ (AIS), and cervical cancer (including both squamous cell carcinoma (SCC) and adenocarcinoma (AC)) [30]. CIN3+ (including CIN3, AIS, and cervical cancer) were defined as the primary endpoint, while CIN2+ as secondary endpoint. Vaginal intraepithelial neoplasia (VaIN) 2 or 3 were classified as CIN 2 or 3.

#### 2.2.1 Statistics

Statistical analysis was performed using SPSS 27.0 software. Three methods were employed to determine the distribution of HPV genotypes [9, 31]: (1) Minimum Estimate (Min.): Considers only women with a single HPV infection; (2) Any Type Estimate (Any.): Counts all instances of HPV infections, with multiple HPV infections being counted repeatedly; (3) Hierarchical Attribution Estimate (Hier.) was based on the (pre)cancer risks of different HPVs, which attribute multiple HPV infections to one specific HPV genotype with the highest risk ranking from Any. Hier.1: Ranks genotypes based on their Any. estimate for CIN2+ cases. Hier.2: Ranks genotypes based on their Any. estimate for CIN3+ cases. *χ* ^2^ tests were used to compare the immediate risk of CIN2+/3+ in LSIL women with HPV‐positive to HPV‐negative (as the control group), with Fisher's exact test employed for parameters with an expected frequency < 5. A *p* value < 0.05 was considered statistically significant, and the *p* values were adjusted for multiple testing using the Benjamini–Hochberg false discovery rate (FDR) at *q* = 0.05. Genotypes with FDR‐corrected *q* < 0.05 were considered statistically significant. The efficacy of conventional and risk‐based management strategies for detecting CIN2+/3+ was evaluated using sensitivity, specificity (ability to identify negatives), positive predictive value (PPV) (ability to identify true positives), negative predictive value (NPV), and the number of colposcopies needed to identify each CIN2+/3+ case. All values were reported with their corresponding 95% confidence intervals (CIs).

## 3 Results

### 3.1 Clinical and Pathological Characteristics of Study Population

**Figure 1:**
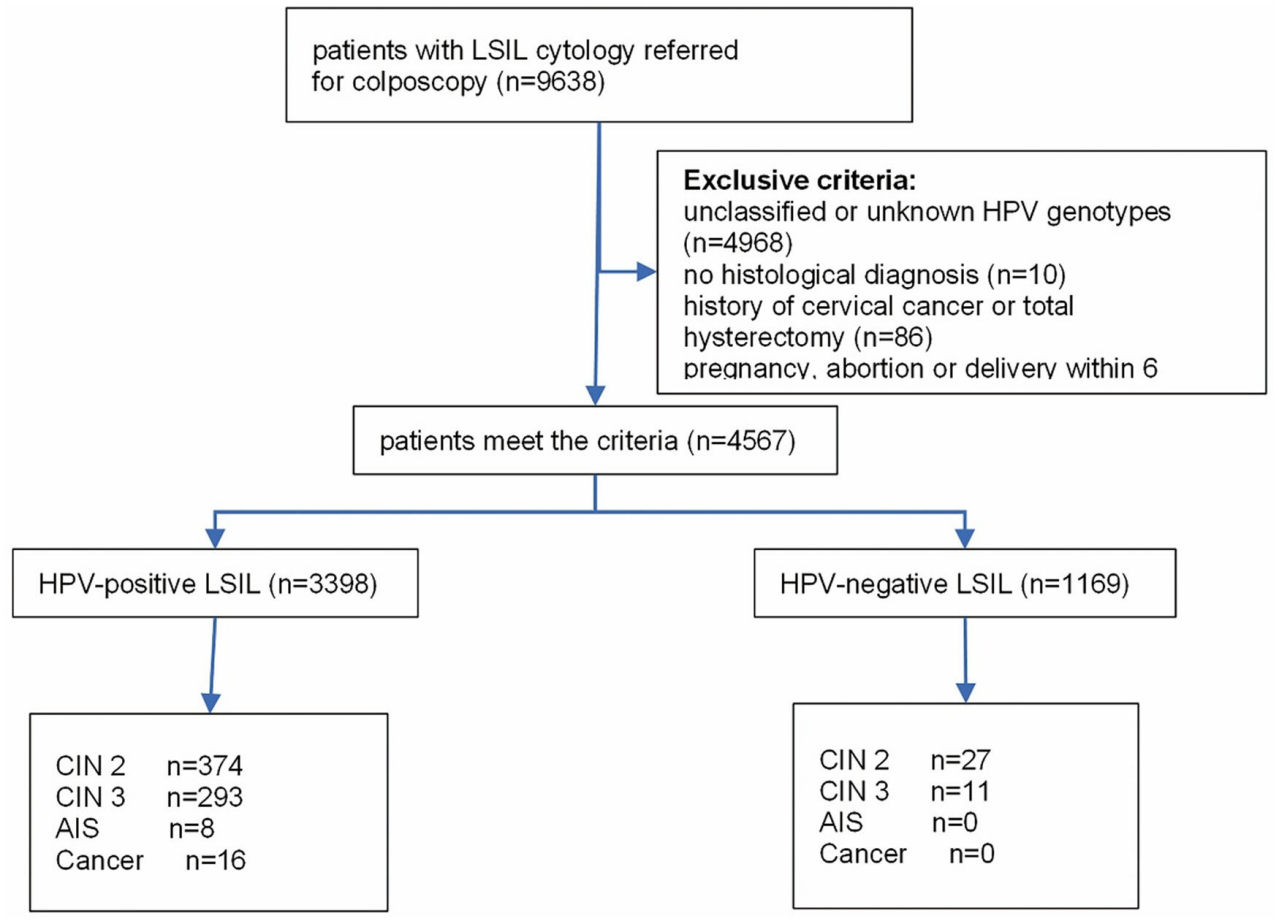
Study flowchart. AIS, adenocarcinoma in situ; CIN, cervical intraepithelial neoplasia; HPV, human papillomavirus; LSIL, low‐grade squamous intraepithelial lesion.

As shown in Figure 1, a total of 4567 patients with LSIL cytology who underwent colposcopy within 3 months were identified. Among them, 3398 (74.40%) tested HPV‐positive, and 1169 (25.60%) tested HPV‐negative. The median age was 40 years (range: 18–75), with 39 years (range: 17–39) for HPV‐positive women and 42 years (range: 19–76) for HPV‐negative women. As shown in Table 1, HPV positive rate varied significantly among different age groups (*χ* ^2^ = 220.45, *p* < 0.0001). The highest rate was observed in women younger than 25 years (83.24%), followed by those aged 25–29 years (81.97%), and a lower rate in the 40–49 years old group (70.02%). This trend may be explained by higher sexual activity among younger women, increasing their risk of HPV exposure. In the HPV‐positive group, 2284 (67.22%) were single infections, while 1114 (32.78%) were multiple infections. The prevalence of single HPV infection was highest in middle‐aged women (30–39 years), while the prevalence of multiple infections was highest in women younger than 25 years.

**Table 1:** Clinical and pathological characteristics of 4567 women with LSIL cytology.

	No. of cases	HPV‐negative (*n* = 1169)	HPV‐positive (*n* = 3398)	Single HPV infection (*n* = 2284)	Multiple HPV infections (*n* = 1114)
Age group, year					
＜ 25	185 (4.05%)	31 (16.76%)	154 (83.24%)	81 (43.78%)	73 (39.46%)
25–29	488 (10.69%)	88 (18.03%)	400 (81.97%)	245 (50.20%)	155 (31.76%)
30–39	1511 (33.09%)	358 (23.69%)	1153 (76.31%)	804 (53.21%)	349 (23.10%)
40–49	1264 (27.68%)	379 (29.98%)	885 (70.02%)	627 49.60%)	258 (20.41%)
50–64	1036 (22.68%)	294 (28.38%)	742 (71.62%)	491 (47.39%)	251 (24.23%)
＞ 65	83 (1.82%)	19 (22.89%)	64 (77.11%)	36 (43.37%)	28 (33.73%)
Pathology					
＜ CIN2	3838 (84.04%)	1131 (96.75%)	2707 (79.66%)	1799 (78.77%)	908 (81.51%)
CIN2	401 (8.78%)	27 (2.31%)	374 (11.01%)	260 (11.38%)	114 (10.23%)
CIN3	304 (6.66%)	11 (0.94%)	293 (8.62%)	205 (8.98%)	88 (7.90%)
AIS	8 (0.18%)	0 (0.00%)	8 (0.24%)	7 (0.31%)	1 (0.09%)
Cancer	16 (0.35%)	0 (0.00%)	16 (0.47%)	13 (0.57%)	3 (0.27%)

Of the 4567 cases, 729 (15.96%) were CIN2+ and 328 (7.18%) were CIN3+ , including 28 VaIN2 and 7 VaIN3 (Table 1). CIN2+ and CIN3+ were 691 (20.34%) and 317 (9.33%) in HPV‐positive women, and 38 (3.25%) and 11 (0.94%) in HPV‐negative women, respectively (*χ* ^2^ = 189.260, *p* < 0.001; *χ* ^2^ = 91.802, *p* < 0.01). The immediate risk of CIN2+/3+ was slightly higher for those with single HPV infections (21.23%/9.85%), compared to those with multiple HPV infections (18.50%/8.26%) (*χ* ^2^ = 3.477, *p* = 0.062; *χ* ^2^ = 2.245, *p* = 0.134) (Table 1). In HPV‐positive group, women aged 30–39 years had the highest risk of CIN2+/3+ , followed by younger women (< 29 years), and the rest decreased with age. In HPV‐negative group, the risk of CIN3+ was lower in all age groups (0%–1.36%), and only older women (≥ 65 years) and middle‐aged women (30–39 years) had a relatively higher risk of CIN2+ (5.26% and 5.03%) (Supporting Information S1: Table S1).

### 3.2 Distribution of HPV Genotypes With LSIL Cytology and Their Proportion in CIN2+/3+ Cases

**Figure 2:**
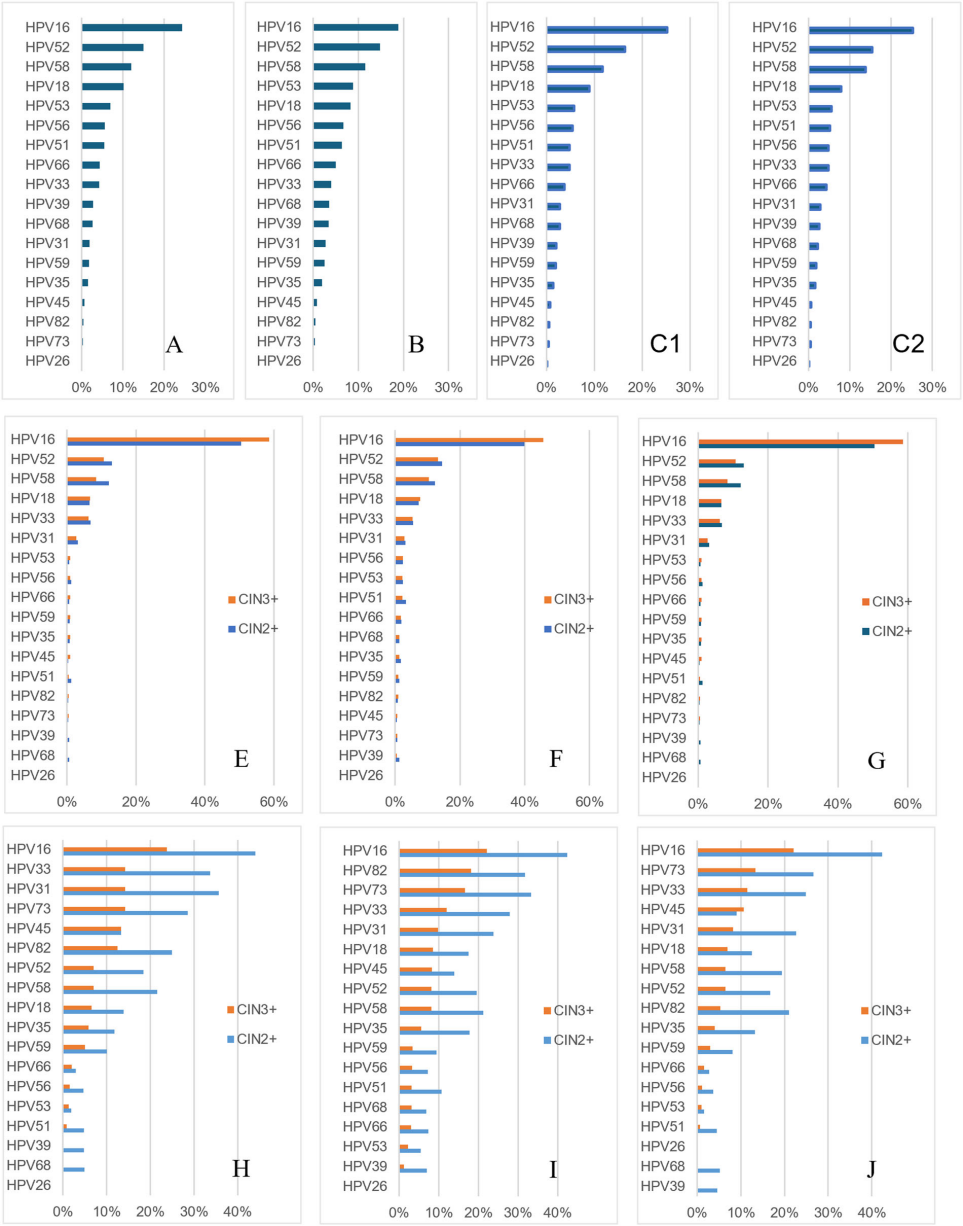
The distribution of specific HPV genotype in HPV‐positive/LSIL women (estimated by Min. (A), by Any. (B), by Hier.1 (C1), by Hier.2 (C2). The distribution of specific HPV genotype in HPV‐positive CIN2+/3+ cases (estimated by Min. (E), by Any. (F), by Hier. (G). The immediate CIN2+/3+ risk of specific HPV genotype in HPV‐positive/LSIL women (estimated by Min. (H), by Any. (I), by Hier. (J). Abbreviations: Any., any type estimate; CIN, cervical intraepithelial neoplasia; Hier., hierarchical attribution estimate; HPV, human papillomavirus; LSIL, low‐grade squamous intraepithelial lesion; Min., minimum estimate.

Three different methods (Min., Any., Hier.) were used to analyze the distribution of specific HPV genotypes among 3398 HPV‐positive LSIL women and their proportion in CIN2+/3+ cases (Supporting Information S1: Table S1). All methods identified HPV16, 52, 58, 18, 53 as the most frequent five genotypes, while HPV26, 73, 82, 45 were the least common (Figure 2A,B,C1,C2). The prevalence order of the remaining nine genotypes differed slightly between the methods. The proportion of all hrHPV infections was higher than that of irHPV infections, except HPV53(up to more than 90% of total hrHPV infections).

HPV16 was the most prevalent genotype associated with both CIN2+ (52.68%) and CIN3+ (59.94%) in HPV‐positive/LSIL women (by Hier.). Other prevalent genotypes associated with CIN2+ included HPV58 (13.17%), HPV52 (12.74%), HPV33 (5.93%), and HPV18 (4.92%), and other prevalent genotypes associated with CIN3+ included HPV52 (11.36%), HPV58 (8.20%), HPV18 (6.62%), and HPV33 (5.99%) successively. Although HPV53 was the fifth most prevalent HPV genotype, it accounted for a low proportion of CIN2+ and CIN3+ cases. (Supporting Information S1: Table S3 and Figure 2E–G).

### 3.3 Immediate Risk of CIN2+/3+ by HPV Genotypes in HPV‐Positive LSIL Women

Our analysis consistently identified HPV16 as the genotype with the highest immediate risk of CIN2+/3+ among HPV‐positive LSIL women, regardless of the calculation method employed. HPV16 (42.37%), HPV73 (26.67%), HPV33 (24.85%), HPV31 (22.68%), HPV82 (21.05%), HPV58 (19.44%), HPV52 (16.76%), HPV35 (13.21%), HPV18 (12.59%), and HPV45 (9.09%) were associated with a high risk of CIN2+ lesions (Hier1.). Respectively, HPV16 (22.12%), HPV73 (13.33%), HPV33 (11.52%), HPV45 (10.71%), HPV31 (8.25%), HPV18 (6.91%), HPV58 (6.50%), HPV52 (6.46%), HPV82 (5.26%), and HPV35 (4.08%) were associated with a high risk of CIN3+ lesions (Hier2.). Several HPV genotypes demonstrated a low risk of progressing to CIN3+ (< 4%), including some hrHPV types (HPV59, 66, 56, 51, 68, and 39), as well as irHPV types HPV53 and HPV26. Notably, while HPV73 and 82 (both irhPVs) were consistently associated with a high risk of CIN 2+/3+, HPV53 and 26 remained consistently low risk. (Supporting Information S1: Table S4 and Figure 2H–J). Among the 16 cancer cases, 15 were infected with HPV16 (12 single infections, 2 co‐infections with HPV18, and 1 co‐infection with HPV52/53), and 1 case was infected with HPV52.

### 3.4 Risk Triage Strategies Based on HPV Genotypes With LSIL Cytology

**Figure 3:**
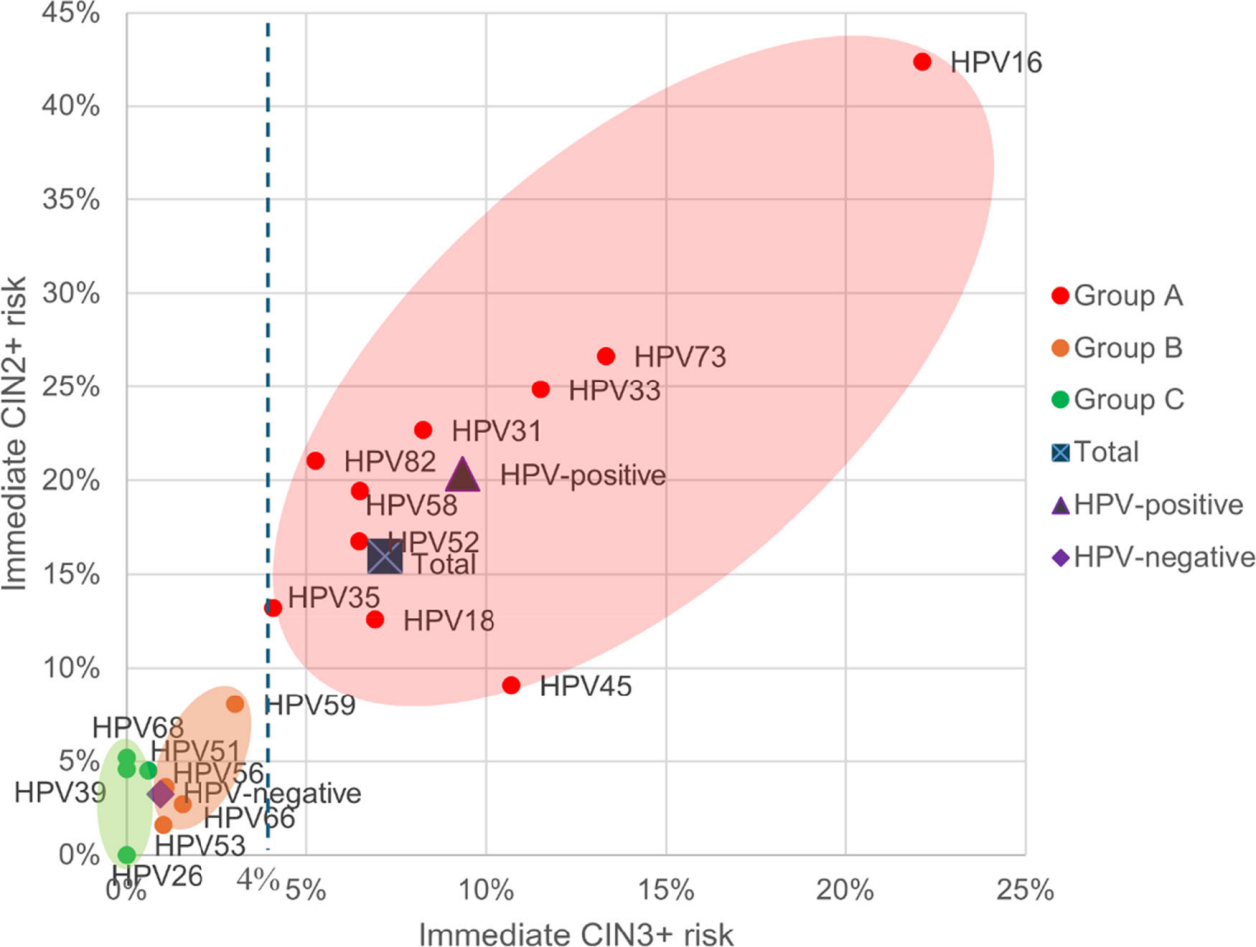
The immediate CIN2+/3+ risk of specific HPV genotype by risk‐based triage strategy. Abbreviations: CIN, cervical intraepithelial neoplasia; HPV, human papillomavirus.

To develop a better strategy for precise management of women with LSIL cytology, the risks associated with different HPV infection statuses were evaluated based on the ASCCP principle of “equal management for equal risk” [23, 24]. Women with HPV‐negative LSIL have a low immediate risk of CIN3+ (less than 4%), so a 1‐year follow‐up was recommended. Immediate CIN2+/3+ risk for each HPV genotype was compared with HPV‐negative group by the Hier. method (Supporting Information S1: Tables S5, and S6). HPV genotypes (16, 33, 45,31, 18, 58, 52, 35, and 73, 82) carried high CIN3+ risk (range, 4.08%–22.12%, all ≥ 4.0%) were classified as group A, and would be referred for immediate colposcopy (Table 2). Genotypes (51, 68, 39, 26) carried low risk (range, 0.00%–0.61%) lower than HPV‐negative LSIL women (0.94%) were categorized as group C, and repeat HPV‐based testing at 1 year is recommended. And the rest genotypes (59, 66, 56, 53) with lower risk (range, 1.03%–3.03%) were classified as group B, and a more closer monitoring (6 months interval) or combination with additional triage methods (p16/Ki‐67 dual stain (DS), DNA methylation detection, etc.) is recommended (Figure 3) [32, 33].

**Table 2:** The risk‐based triage strategy based on HPV genotype with LSIL cytology.

		Group	*N*	Immediate risk, *n*%	Management
CIN 3+	A	16/73/33/45/31/18/58/52/82/35	2493	308 (12.35)	Colposcopy
	B	59/66/56/53	601	8 (1.33)	6 months follow‐up or other triage tests
	C	51/68/39/26	304	1 (0.33)	1‐year follow‐up as HPV‐negative

As the conventional strategy, all HPV‐positive LSIL women were referred for colposcopy. However, our new risk‐based triage strategy recommends immediate colposcopy only for LSIL women infected with HPV genotypes in group A. Both strategies demonstrated high sensitivity and negative predictive value (NPV) in detecting CIN2+/3+ lesions. The risk‐based strategy showed significantly higher specificity (CIN2+: 29.47% vs. 52.16%, *χ* ^2^ = 409.136, *p* < 0.001; CIN3+: 27.32% vs. 48.45%, *χ* ^2^ = 402.395, *p* < 0.001) and positive predictive value (PPV) (CIN2+: 20.34% vs. 26.35%, *χ* ^2^ = 29.515, *p* < 0.001; CIN3+: 9.33% vs. 12.35%, *χ* ^2^ = 13.88, *p* < 0.001) (Table 3). In addition, this strategy could also reduce the number of colposcopies required to detect each CIN2+/3+ lesion (CIN2+: 4.92 vs. 3.79, *χ* ^2^ = 29.515, *p* < 0.001; CIN3+: 10.72 vs. 8.09, *χ* ^2^ = 13.88, *p* < 0.001).

**Table 3:** Efficacy of conventional strategy and risk‐based triage strategy.

		Sensitivity (% [95% CI])	Specificity (% [95% CI])	PPV (% [95% CI])	NPV (% [95% CI])	*N*/colposcopy
Conventional						
	CIN2+	94.79 (92.85–96.24)	29.47 (28.03–30.94)	20.34 (19.00–21.74)	96.75 (95.52–97.66)	4.92
	CIN3+	96.65 (93.90–98.23)	27.32 (25.99–28.69)	9.33 (8.38–10.37)	99.06 (98.27–99.50)	10.72
Risk‐based						
	CIN2+	90.12(87.67–92.15)	52.16 (50.57–53.75)	26.35 (24.64–28.14)	96.53 (95.62–97.26)	3.79
	CIN3+	93.90 (90.59–96.14)	48.45 (46.94–49.97)	12.35 (11.10–13.73)	99.04 (98.49–99.39)	8.09

**Figure 4:**
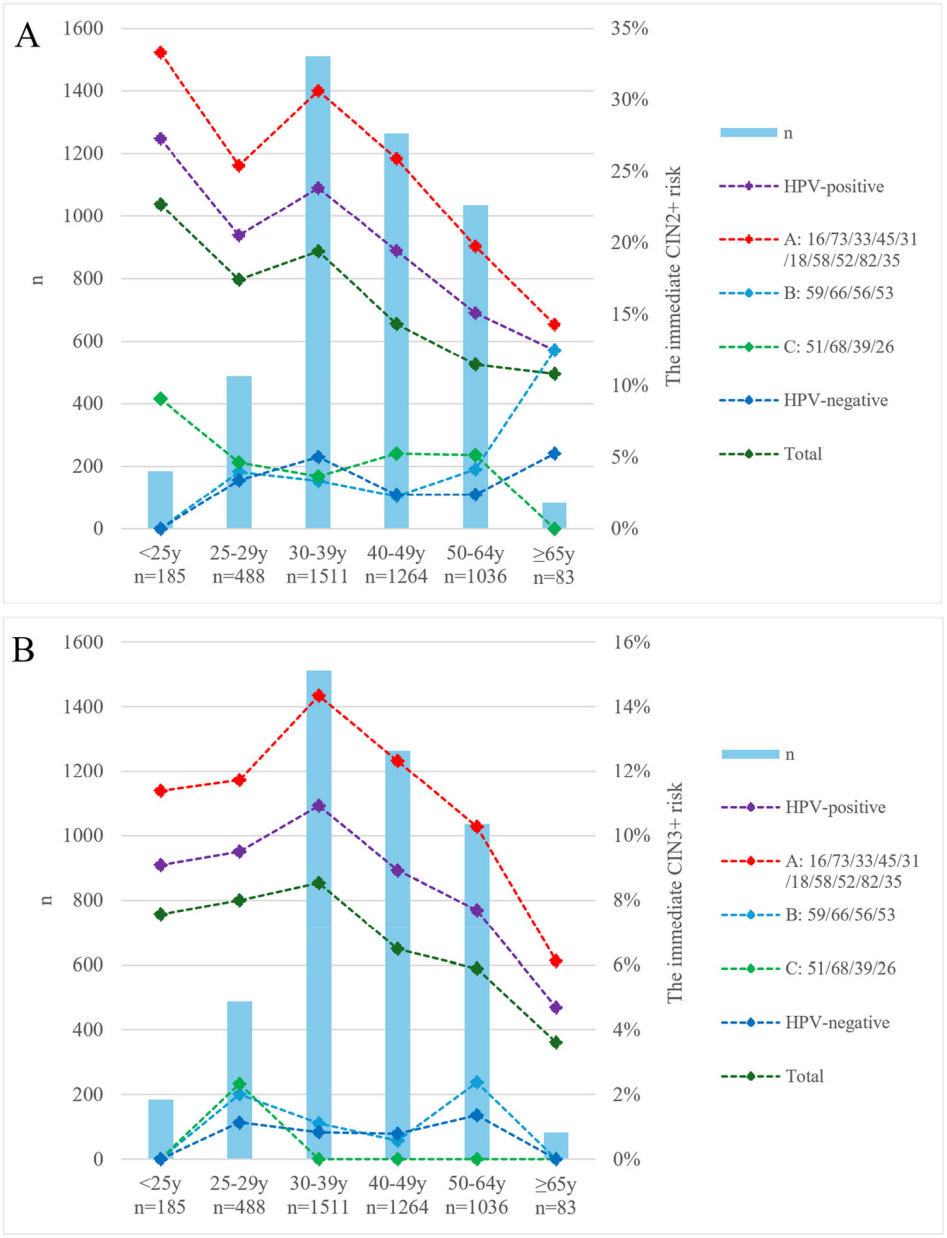
The immediate CIN2+ (A) and CIN3+ (B) risk of HPV‐positive women, three risk‐stratified groups, HPV‐negative women, and the total population stratified by age. CIN, cervical intraepithelial neoplasia; HPV, human papillomavirus.

We further analyzed the association between different HPV types and CIN2+/3+ risk stratified by age (Figure 4). After age 30, there was a decreasing trend in CIN2+/3+ risk with age. The results showed a general decrease in CIN2+/3+ risk after age 30. Group A consistently exhibited the highest risk of CIN2+ and CIN3+, while group B and C showed varying risks across age groups. Notably, younger women (< 25 years) infected with group C and older women (≥ 65 years) infected with group B had higher CIN2+ risk. This age‐related difference may be influenced by factors such as immune response and lifestyle. For young women (< 25 years), given the high prevalence of HPV infection and the high rate of spontaneous CIN2 regression, individualized follow‐up management is recommended, considering the ASCCP guidelines and individual fertility needs.

## 4 Discussion

With increased HPV vaccination, accurate risk assessment for non‐16/18 HPV genotypes becomes increasingly important, and extended genotyping enables it to be possible [24, 34]. This study is the first to comprehensively describe the risk of both CIN2+ and CIN3+ associated with 18 specific high‐ and intermediate‐risk HPV types in a large Chinese female population with LSIL cytology, providing a scientific basis for more precise risk stratification and refined management in cervical cancer screening. Organized cervical cancer screening is usually government‐funded in most countries, including China. Our results suggested extended HPV genotyping could improve cost‐effectiveness, no matter whether it serves as a primary screening tool or is combined with cytology, which would greatly reduce the economic burden, particularly in low‐income and middle‐income countries (LMICs).

Previous studies reported a wide range of HPV infection rates (61%–83%) in women with LSIL cytology, varying by region [16, 18, 20]. Our study found a 74.4% prevalence rate, similar to that of a large CAP‐certified laboratory in China [16]. Interestingly, the total HPV infection rates showed a U‐shaped distribution by age, with higher rates in younger (< 25 years) and older (≥ 65 years) women, suggesting complex factors with HPV infection, including age, sexual behavior, and immune function. The five most common HPV types in the general female population were HPV16, 18, 31, 58, and 52, according to WHO worldwide data, while HPV16, 52, 58, 18, and 33 were found in 1.7 million Chinese individuals [35]. In a study of 4328 Chinese women with LSIL cytology, Tao et al. found HPV52, 53, 16 were the most prevalent genotypes, and HPV16, 82, 33, 31, 26 were most frequently associated with CIN2+ lesions [28]. Regardless of the calculation method used, our data showed HPV16, 52, 58, 18, and 53 were the five most prevalent, while HPV26, 73, 82, and 45 were less frequently found among LSIL women, consistent with prior findings [28]. HPV16, 52, 58, 18, and 33 were most common in (pre)cancer. Notably, HPV53, despite its high infective prevalence, was less frequent in CIN2+ and CIN3+ cases, suggesting lower carcinogenic risk [5]. These findings highlight the importance of considering a wide spectrum of hrHPV and intermediate‐risk HPV types in CIN2+/3+ risk assessment.

As anticipated, women with HPV‐positive LSIL in our study showed a higher immediate risk of CIN2+/3+ (20.34%/9.33%) compared to those with HPV‐negative LSIL (3.25%/0.94%). Although colposcopy is typically recommended for all HPV‐positive women with LSIL, the risk of CIN2+/3+ varied across HPV genotypes [13, 25, 28, 29]. Similar to these findings, our study identified HPV16, 73, 33, 31, 82, 58, 52, 35, and 18 carried the highest risk for CIN2+ (range: 12.59%–42.37%). However, prior studies mainly focused on CIN2+. Given the inherent diagnostic challenges and potential for spontaneous regression of CIN2, CIN3 is a more clinically relevant endpoint for risk assessment with reproducible diagnosis. The Onclarity trial evaluating 854 women with LSIL, found HPV16 with the highest CIN3+ risk (13.0%), followed by HPV31, 18, 45, and 33/58 (1.9%–6.5%), while HPV56/59/66 showed no detectable risk [25]. However, it grouped certain HPV genotypes, which might mask the risk variations between specific types. In addition to the similar findings of hrHPV genotypes, our study further observed an increased risk of CIN2+/3+ for HPV73 and 82. However, Tao et al. observed a higher CIN2+ risk for HPV26 and 82, which might be attributed to an insufficient evaluation of rare HPV genotypes [28]. While the updated extended HPV genotyping guideline classified HPV 59/56/66 as low risk [34], our data found 1.08%–3.03% CIN3+ risk for these HPV types in LSIL cytology (as Group B), suggesting closer follow‐up or further triage. This discrepancy may reflect regional variations in their carcinogenic potential.

As mentioned above, extended genotyping improves risk stratification in cervical cancer screening. In a study of 902 Chinese women with LSIL, Xue et al. showed that a genotyping model including HPV 16/18/31/33/52/58 improved CIN2+ detection sensitivity (92.9% vs. 54.9%) but lower specificity (31.4% vs. 79.6%) compared to HPV16/18 alone, while maintaining high NPV (93.0%) with a 25.3% reduction in referrals [29]. Our risk‐based triage strategy, referring only 10 HPV types with a CIN3+ risk > 4.0% for colposcopy, yielded significantly higher specificity (CIN2+: 52.16% vs. 29.47%, CIN3+: 48.45% vs. 27.32%, *p* < 0.001) and PPV (CIN2+: 26.35% vs. 20.34%, CIN3+: 12.35% vs. 9.33%, *p* < 0.001) compared to the conventional strategy. Importantly, this approach reduced immediate colposcopy referrals by 19.82% while maintaining high sensitivity (CIN2+: 90.12%, CIN3+: 93.90%) and NPV (CIN2+: 96.53%, CIN3+: 99.04%). Thus, our risk‐based strategy may offer potential clinical benefits for women with LSIL cytology by improving treatment and follow‐up decisions. Considering the higher immediate CIN2+ risk associated with Group B HPV types in women aged ≥ 65 years, it suggests a potential rationale for HPV screening in this older demographic (over 65 years old). However, further research is warranted.

With the development of molecular detection techniques, recent p16/Ki‐67 DS and DNA methylation are being used for better triage management of hrHPV‐positive women [35, 36]. The prospective PALMS study demonstrated comparable sensitivity of p16/Ki‐67 DS (CIN2+: 85.7% vs. 98.4%, *p* = 0.005; CIN3+: 88.0% vs. 100%, *p* = 0.084), but significantly higher specificity (CIN2+: 53.3% vs. 15.6%, *p* < 0.001; CIN3+: 49.3% vs. 14.2%, *p* < 0.001) for diseases detection in LSIL cytology, when compared to HC2 testing [37]. Our study's colposcopy referral rate (54.59%) showed similar specificity and potentially higher sensitivity than PALMS. Using test performance data from the KPNC study, cotesting followed by p16/Ki‐67 DS triage results in 11% fewer total colposcopies and 64% fewer years to CIN3+ diagnoses compared to cotesting alone [32]. DNA methylation offers high specificity as a complement triage test to hrHPV [33, 36], with high sensitivity and specificity to triage hr‐HPV positive women (CIN2+: 56.7% and 87.6%, CIN3+: 66.7% and 84.1%) [38]. Therefore, for group B HPVs (59/66/56/53) in our study, triage efficacy combined with DS or DNA methylation detection is worthy of further study. Furthermore, based on cost‐effectiveness, routine use of extended genotyping for further triage of HPV‐positive women is not currently recommended [35].

Nevertheless, the present study had some limitations. As a retrospective study, it was subject to selection bias, and findings may not fully represent other populations due to geographical variations. Small sample sizes for HPV73, HPV82, and HPV26 infections made it difficult to accurately assess their pathogenic risks. Current HPV assays, including the one used, may also have issues with viral gene fragment loss, necessitating more robust detection methods. Our study lacked a large‐scale follow‐up, which will be addressed in future research endeavors. The successful implementation of risk‐based cervical cancer screening methods demands careful consideration of local medical resources, patient preferences, and healthcare provider proficiency. Future research should explore the feasibility and effectiveness of this approach across different settings.

## 5 Conclusion

The present study stratified 14 hrHPV and 4 intermediate‐risk HPV types into three groups based on the immediate risk of CIN3+ among LSIL in accordance with the risk thresholds of ASCCP guidelines. Compared with the conventional management strategy for LSIL, this new risk‐based stratification strategy exhibited that it can not only effectively reduce unnecessary colposcopies but also maintain high efficacy for CIN2+/3+ detection. However, more comprehensive studies are still needed globally. In the future, we will validate these findings in a large‐scale cervical cancer screening cohort to provide more evidence on the cumulative risk of specific HPV types among LSIL women, to fully assess the long‐term effects and feasibility of widespread implementation of this strategy.

## Author Contributions

**Chun Ye:** methodology, conceptualization, data curation, software, validation, writing – original draft, writing – review and editing. **Yi Liu:** data curation, methodology, and software. **Huiru Huang:** investigation and software. **Ruizhe Chen:** data curation and software. **Ying Li:** data curation. **XiaoFei Zhang:** data curation. **Yunfeng Fu:** data curation, investigation, and validation. **Liang Feng:** writing – review and editing, project administration, and resources. **Xiao Li:** conceptualization, writing – review and editing, project administration, and resources.

## Disclosure

The authors alone are responsible for the views expressed in this paper, and they do not necessarily represent the views, decisions, or policies of the institutions with which they are affiliated.

## Ethics Statement

This study protocol was approved by the ethics committee of the Women's hospital school of Medicine Zhejiang University (IRB‐20240280‐R). Owing to the retrospective character of the study, informed consent was specifically waived by the ethics committee.

## Conflicts of Interest

The authors declare no conflicts of interest.

## Supporting information

Supplementary table ‐Risk‐based Triage Strategy by Extended HPV Genotyping for Women with LSIL Cytology‐JMV0422.

## Data Availability

The data that support the findings of this study are available on request from the corresponding author. The data are not publicly available due to privacy or ethical restrictions.
