# Supplementary material for: Risk‐Based Triage Strategy by Extended HPV Genotyping for Women With LSIL Cytology: A Real‐World Study
Source: J Med Virol. 2025 May 20;97(5):e70404. doi: 10.1002/jmv.70404 (PMC12090974; doi:10.1002/jmv.70404)
Supplement: Supplementary file 1 — Supplementary table ‐Risk‐based Triage Strategy by Extended HPV Genotyping for Women with LSIL Cytology‐JMV0422. [file JMV-97-e70404-s001.docx]

Supplementary Table S1. Pathological characteristics of HPV-positive vs. HPV-negative by age groups.

| **Age Group, year** |  | **No. of cases** |  | **＜CIN2** |  | **CIN2** |  | **CIN3** |  | **AIS** |  | **CC** |
| --- | --- | --- | --- | --- | --- | --- | --- | --- | --- | --- | --- | --- |
| **HPV-positive** |  |  |  |  |  |  |  |  |  |  |  |  |
| ＜25 |  | 154 |  | 112 |  | 28 |  | 11 |  | 3 |  | 0 |
| 25-29 |  | 400 |  | 318 |  | 44 |  | 37 |  | 1 |  | 0 |
| 30-39 |  | 1153 |  | 878 |  | 149 |  | 126 |  | 0 |  | 0 |
| 40-49 |  | 885 |  | 713 |  | 93 |  | 71 |  | 2 |  | 6 |
| 50-64 |  | 742 |  | 630 |  | 55 |  | 46 |  | 2 |  | 9 |
| ≥65 |  | 64 |  | 56 |  | 5 |  | 2 |  | 0 |  | 1 |
| **HPV-negative** |  |  |  |  |  |  |  |  |  |  |  |  |
| ＜25 |  | 31 |  | 31 |  | 0 |  | 0 |  | 0 |  | 0 |
| 25-29 |  | 88 |  | 85 |  | 2 |  | 1 |  | 0 |  | 0 |
| 30-39 |  | 358 |  | 340 |  | 15 |  | 3 |  | 0 |  | 0 |
| 40-49 |  | 379 |  | 370 |  | 6 |  | 3 |  | 0 |  | 0 |
| 50-64 |  | 294 |  | 287 |  | 3 |  | 4 |  | 0 |  | 0 |
| ≥65 |  | 19 |  | 18 |  | 1 |  | 0 |  | 0 |  | 0 |

Supplementary Table S2. The distribution of specific 18 HPV genotypes among 3398 HPV-positive LSIL women

| **Min. (n=2284)** | |  | **Any. (n=4565)** | |  | **Hier.1 (n=3398)** | |  | **Hier.2 (n=3398)** | | |
| --- | --- | --- | --- | --- | --- | --- | --- | --- | --- | --- | --- |
| HPV16 | 556 (24.34%) |  | HPV16 | 859 (18.82%) |  | HPV16 | 859 (25.28%) |  | HPV16 | 859 (25.28%) | |
| HPV52 | 341 (14.93%) |  | HPV52 | 674 (14.76%) |  | HPV52 | 525 (15.45%) |  | HPV52 | 557 (16.39%) | |
| HPV58 | 273 (11.95%) |  | HPV58 | 528 (11.57%) |  | HPV58 | 468 (13.77%) |  | HPV58 | 400 (11.77%) | |
| HPV18 | 231 (10.11%) |  | HPV53 | 400 (8.76%) |  | HPV18 | 270 (7.95%) |  | HPV18 | 304 (8.95%) | |
| HPV53 | 158 (6.92%) |  | HPV18 | 376 (8.24%) |  | HPV53 | 188 (5.53%) |  | HPV53 | 195 (5.74%) | |
| HPV56 | 129 (5.65%) |  | HPV56 | 305 (6.68%) |  | HPV51 | 178 (5.24%) |  | HPV56 | 185 (5.44%) | |
| HPV51 | 126 (5.52%) |  | HPV51 | 289 (6.33%) |  | HPV33 | 165 (4.86%) |  | HPV33 | 165 (4.86%) | |
| HPV66 | 101 (4.42%) |  | HPV66 | 229 (5.02%) |  | HPV56 | 165 (4.86%) |  | HPV51 | 165 (4.86%) | |
| HPV33 | 98 (4.29%) |  | HPV33 | 183 (4.01%) |  | HPV66 | 147 (4.33%) |  | HPV66 | 128 (3.77%) | |
| HPV39 | 63 (2.76%) |  | HPV68 | 161 (3.53%) |  | HPV31 | 97 (2.85%) |  | HPV31 | 97 (2.85%) | |
| HPV68 | 61 (2.67%) |  | HPV39 | 156 (3.42%) |  | HPV39 | 87 (2.56%) |  | HPV68 | 94 (2.77%) | |
| HPV31 | 42 (1.84%) |  | HPV31 | 122 (2.67%) |  | HPV68 | 77 (2.27%) |  | HPV39 | 71 (2.09%) | |
| HPV59 | 40 (1.75%) |  | HPV59 | 116 (2.54%) |  | HPV59 | 62 (1.82%) |  | HPV59 | 66 (1.94%) | |
| HPV35 | 34 (1.49%) |  | HPV35 | 90 (1.97%) |  | HPV35 | 53 (1.56%) |  | HPV35 | 49 (1.44%) | |
| HPV45 | 15 (0.66%) |  | HPV45 | 36 (0.79%) |  | HPV45 | 22 (0.65%) |  | HPV45 | 28 (0.82%) | |
| HPV82 | 8 (0.35%) |  | HPV82 | 22 (0.48%) |  | HPV82 | 19 (0.56%) |  | HPV82 | 19 (0.56%) |  |
| HPV73 | 7 (0.31%) |  | HPV73 | 18 (0.39%) |  | HPV73 | 15 (0.44%) |  | HPV73 | 15 (0.44%) |  |
| HPV26 | 1 (0.04%) |  | HPV26 | 1 (0.02%) |  | HPV26 | 1 (0.03%) |  | HPV26 | 1 (0.03%) |  |

Supplementary Table S3. The proportion of HPV genotypes in HPV-positive LSIL CIN2+/3+ cases (by Min., Any. and Hier.)

| **Min.** | | | | |  | **Any.** | | | | |  | **Hier.1** | |  | **Hier.2** | |
| --- | --- | --- | --- | --- | --- | --- | --- | --- | --- | --- | --- | --- | --- | --- | --- | --- |
| **CIN2+** | |  | **CIN3+** | |  | **CIN2+** | |  | **CIN3+** | |  | **CIN2+** | |  | **CIN3+** | |
| HPV16 | 245 (50.52%) |  | HPV16 | 132 (58.67%) |  | HPV16 | 364 (39.87%) |  | HPV16 | 190 (45.78%) |  | HPV16 | 364 (52.68%) |  | HPV16 | 190 (59.94%) |
| HPV52 | 63 (12.99%) |  | HPV52 | 24 (10.67%) |  | HPV52 | 132 (14.46%) |  | HPV52 | 55 (13.25%) |  | HPV58 | 91 (13.17%) |  | HPV52 | 36 (11.36%) |
| HPV58 | 59 (12.16%) |  | HPV58 | 19 (8.44%) |  | HPV58 | 112 (12.27%) |  | HPV58 | 43 (10.36%) |  | HPV52 | 88 (12.74%) |  | HPV58 | 26 (8.20%) |
| HPV33 | 33 (6.80%) |  | HPV18 | 15 (6.67%) |  | HPV18 | 66 (7.23%) |  | HPV18 | 32 (7.71%) |  | HPV33 | 41 (5.93%) |  | HPV18 | 21 (6.62%) |
| HPV18 | 32 (6.60%) |  | HPV33 | 14 (6.22%) |  | HPV33 | 51 (5.59%) |  | HPV33 | 22 (5.30%) |  | HPV18 | 34 (4.92%) |  | HPV33 | 19 (5.99%) |
| HPV31 | 15 (3.09%) |  | HPV31 | 6 (2.67%) |  | HPV51 | 31 (3.40%) |  | HPV31 | 12 (2.89%) |  | HPV31 | 22 (3.18%) |  | HPV31 | 8 (2.52%) |
| HPV51 | 6 (1.24%) |  | HPV56 | 2 (0.89%) |  | HPV31 | 29 (3.18%) |  | HPV56 | 10 (2.41%) |  | HPV51 | 8 (1.16%) |  | HPV45 | 3 (0.95%) |
| HPV56 | 6 (1.24%) |  | HPV35 | 2 (0.89%) |  | HPV56 | 22 (2.41%) |  | HPV51 | 9 (2.17%) |  | HPV35 | 7 (1.01%) |  | HPV73 | 2 (0.63%) |
| HPV35 | 4 (0.82%) |  | HPV59 | 2 (0.89%) |  | HPV53 | 22 (2.41%) |  | HPV53 | 9 (2.17%) |  | HPV56 | 6 (0.87%) |  | HPV35 | 2 (0.63%) |
| HPV59 | 4 (0.82%) |  | HPV66 | 2 (0.89%) |  | HPV66 | 17 (1.86%) |  | HPV66 | 7 (1.69%) |  | HPV59 | 5 (0.72%) |  | HPV59 | 2 (0.63%) |
| HPV68 | 3 (0.62%) |  | HPV53 | 2 (0.89%) |  | HPV35 | 16 (1.75%) |  | HPV35 | 5 (1.20%) |  | HPV73 | 4 (0.58%) |  | HPV56 | 2 (0.63%) |
| HPV39 | 3 (0.62%) |  | HPV45 | 2 (0.89%) |  | HPV39 | 11 (1.20%) |  | HPV68 | 5 (1.20%) |  | HPV82 | 4 (0.58%) |  | HPV66 | 2 (0.63%) |
| HPV66 | 3 (0.62%) |  | HPV51 | 1 (0.44%) |  | HPV59 | 11 (1.20%) |  | HPV59 | 4 (0.96%) |  | HPV66 | 4 (0.58%) |  | HPV53 | 2 (0.63%) |
| HPV53 | 3 (0.62%) |  | HPV73 | 1 (0.44%) |  | HPV68 | 11 (1.20%) |  | HPV82 | 4 (0.96%) |  | HPV39 | 4 (0.58%) |  | HPV82 | 1 (0.32%) |
| HPV73 | 2 (0.41%) |  | HPV82 | 1 (0.44%) |  | HPV82 | 7 (0.77%) |  | HPV73 | 3 (0.72%) |  | HPV68 | 4 (0.58%) |  | HPV51 | 1 (0.32%) |
| HPV82 | 2 (0.41%) |  | HPV68 | 0 (0.00%) |  | HPV73 | 6 (0.66%) |  | HPV45 | 3 (0.72%) |  | HPV53 | 3 (0.43%) |  | HPV68 | 0 (0.00%) |
| HPV45 | 2 (0.41%) |  | HPV39 | 0 (0.00%) |  | HPV45 | 5 (0.55%) |  | HPV39 | 2 (0.48%) |  | HPV45 | 2 (0.29%) |  | HPV39 | 0 (0.00%) |
| HPV26 | 0 (0.00%) |  | HPV26 | 0 (0.00%) |  | HPV26 | 0 (0.00%) |  | HPV26 | 0 (0.00%) |  | HPV26 | 0 (0.00%) |  | HPV26 | 0 (0.00%) |

Supplementary Table S4. Immediate risk of CIN2+/3+ by specific HPV genotype (by Min., Any., and Hier.)

| **Min.** | | | |  | **Any.** | | | |  | **Hier.** | | | |
| --- | --- | --- | --- | --- | --- | --- | --- | --- | --- | --- | --- | --- | --- |
|  | **CIN2+** |  | **CIN3+** |  |  | **CIN2+** |  | **CIN3+** |  |  | **Hier.1 CIN2+** |  | **Hier.2 CIN3+** |
| HPV16 | 245 (44.06%) |  | 132 (23.74%) |  | HPV16 | 364 (42.37%) |  | 190 (22.12%) |  | HPV16 | 364 (42.37%) |  | 190 (22.12%) |
| HPV31 | 15 (35.71%) |  | 6 (14.29%) |  | HPV82 | 7 (31.82%) |  | 4 (18.18%) |  | HPV73 | 4 (26.67%) |  | 2 (13.33%) |
| HPV33 | 33 (33.67%) |  | 14 (14.29%) |  | HPV73 | 6 (33.33%) |  | 3 (16.67%) |  | HPV33 | 41 (24.85%) |  | 19 (11.52%) |
| HPV73 | 2 (28.57%) |  | 1 (14.29%) |  | HPV33 | 51 (27.87%) |  | 22 (12.02%) |  | HPV45 | 2 (9.09%) |  | 3 (10.71%) |
| HPV45 | 2 (13.33%) |  | 2 (13.33%) |  | HPV31 | 29 (23.77%) |  | 12 (9.84%) |  | HPV31 | 22 (22.68%) |  | 8 (8.25%) |
| HPV82 | 2 (25.00%) |  | 1 (12.50%) |  | HPV18 | 66 (17.55%) |  | 32 (8.51%) |  | HPV18 | 34 (12.59%) |  | 21 (6.91%) |
| HPV52 | 63 (18.48%) |  | 24 (7.04%) |  | HPV45 | 5 (13.89%) |  | 3 (8.33%) |  | HPV58 | 91 (19.44%) |  | 26 (6.50%) |
| HPV58 | 59 (21.61%) |  | 19 (6.96%) |  | HPV52 | 132 (19.58%) |  | 55 (8.16%) |  | HPV52 | 88 (16.76%) |  | 36 (6.46%) |
| HPV18 | 32 (13.85%) |  | 15 (6.49%) |  | HPV58 | 112 (21.21%) |  | 43 (8.14%) |  | HPV82 | 4 (21.05%) |  | 1 (5.26%) |
| HPV35 | 4 (11.76%) |  | 2 (5.88%) |  | HPV35 | 16 (17.78%) |  | 5 (5.56%) |  | HPV35 | 7 (13.21%) |  | 2 (4.08%) |
| HPV59 | 4 (10.00%) |  | 2 (5.00%) |  | HPV59 | 11 (9.48%) |  | 4 (3.45%) |  | HPV59 | 5 (8.06%) |  | 2 (3.03%) |
| HPV66 | 3 (2.97%) |  | 2 (1.98%) |  | HPV56 | 22 (7.21%) |  | 10 (3.28%) |  | HPV66 | 4 (2.72%) |  | 2 (1.56%) |
| HPV56 | 6 (4.65%) |  | 2 (1.55%) |  | HPV51 | 31 (10.73%) |  | 9 (3.11%) |  | HPV56 | 6 (3.64%) |  | 2 (1.08%) |
| HPV53 | 3 (1.90%) |  | 2 (1.27%) |  | HPV68 | 11 (6.83%) |  | 5 (3.11%) |  | HPV53 | 3 (1.60%) |  | 2 (1.03%) |
| HPV51 | 6 (4.76%) |  | 1 (0.79%) |  | HPV66 | 17 (7.42%) |  | 7 (3.06%) |  | HPV51 | 8 (4.49%) |  | 1 (0.61%) |
| HPV39 | 3 (4.76%) |  | 0 (0.00%) |  | HPV53 | 22 (5.50%) |  | 9 (2.25%) |  | HPV68 | 4 (5.19%) |  | 0 (0.00%) |
| HPV68 | 3 (4.92%) |  | 0 (0.00%) |  | HPV39 | 11 (7.05%) |  | 2 (1.28%) |  | HPV39 | 4 (4.60%) |  | 0 (0.00%) |
| HPV26 | 0 (0.00%) |  | 0 (0.00%) |  | HPV26 | 0 (0.00%) |  | 0 (0.00%) |  | HPV26 | 0 (0.00%) |  | 0 (0.00%) |

Supplementary Table S5. The immediate CIN2+ risk with different HPV genotypes in LSIL women (by Hier1.)

|  |  | **Histopathology** | | |  | **CIN2+ Risk** |  | **χ2** |  | **OR** |  | **95%CI** |  | **P-value** |  | **Adj. Q-value** |  | **Sig. (q<0.05)** |
| --- | --- | --- | --- | --- | --- | --- | --- | --- | --- | --- | --- | --- | --- | --- | --- | --- | --- | --- |
|  |  | **CIN2+** |  | **<CIN2** |  |  |  |  |  |  |  |  |  |  |  |  |  |  |
| **HPV-positive(n=3398)** |  | 691 |  | 2707 |  | 20.34% |  | 189.26 |  | 7.597 |  | 5.441-10.609 |  | <0.001 |  |  |  |  |
| **HPV-negative(n=1169)** |  | 38 |  | 1131 |  | 3.25% |  |  |  |  |  |  |  |  |  |  |  |  |
| **18 HPV Genotypes** |  |  |  |  |  |  |  |  |  |  |  |  |  |  |  |  |  |  |
| HPV16(n=859) |  | 364 |  | 495 |  | 42.37% |  | 476.89 |  | 21.886 |  | 15.416-31.072 |  | <0.001 |  | <0.001 |  | **★** |
| HPV73(n=15) |  | 4 |  | 11 |  | 26.67% |  |  |  | 10.823 |  | 3.295-35.546 |  | 0.001 |  | 0.012 |  | **★** |
| HPV33(n=165) |  | 41 |  | 124 |  | 24.85% |  | 121.06 |  | 9.841 |  | 6.097-15.883 |  | <0.001 |  | <0.001 |  | **★** |
| HPV31(n=97) |  | 22 |  | 75 |  | 22.68% |  | 74.896 |  | 8.731 |  | 4.914-15.511 |  | <0.001 |  | 0.002 |  | **★** |
| HPV82(n=19） |  | 4 |  | 15 |  | 21.05% |  |  |  | 7.937 |  | 2.515-25.049 |  | 0.004 |  | 0.036 |  | **★** |
| HPV58(n=468) |  | 91 |  | 377 |  | 19.44% |  | 120.73 |  | 7.184 |  | 4.835-10.676 |  | <0.001 |  | <0.001 |  | **★** |
| HPV52(n=525) |  | 88 |  | 437 |  | 16.76% |  | 96.064 |  | 5.993 |  | 4.033-8.907 |  | <0.001 |  | <0.001 |  | **★** |
| HPV35(n=53) |  | 7 |  | 46 |  | 13.21% |  | 11.503 |  | 4.529 |  | 1.92-10.686 |  | <0.001 |  | 0.004 |  | **★** |
| HPV18(n=270) |  | 34 |  | 236 |  | 12.59% |  | 40.273 |  | 4.288 |  | 2.644-6.954 |  | <0.001 |  | 0.003 |  | **★** |
| HPV45(n=22) |  | 2 |  | 20 |  | 9.09% |  |  |  | 2.976 |  | 0.671-13.194 |  | 0.167 |  | 0.201 |  | - |
| HPV59(n=62) |  | 5 |  | 57 |  | 8.06% |  | 2.745 |  | 2.611 |  | 0.99-6.885 |  | 0.098 |  | 0.147 |  | - |
| HPV68(n=77) |  | 4 |  | 73 |  | 5.19% |  | 0.348 |  | 1.631 |  | 0.567-4.694 |  | 0.555 |  | 0.555 |  | - |
| HPV39(n=87) |  | 4 |  | 83 |  | 4.60% |  | 0.133 |  | 1.434 |  | 0.5-4.116 |  | 0.715 |  | 0.715 |  | - |
| HPV51(n=178) |  | 8 |  | 170 |  | 4.49% |  | 0.724 |  | 1.401 |  | 0.643-3.053 |  | 0.395 |  | 0.395 |  | - |
| HPV56(n=165） |  | 6 |  | 159 |  | 3.64% |  | 0.067 |  | 1.123 |  | 0.467-2.699 |  | 0.795 |  | 0.795 |  | - |
| HPV66(n=147) |  | 4 |  | 143 |  | 2.72% |  | 0.009 |  | 0.833 |  | 0.293-2.367 |  | 0.924 |  | 0.924 |  | - |
| HPV53(n=188) |  | 3 |  | 185 |  | 1.60% |  | 1.514 |  | 0.483 |  | 0.147-1.58 |  | 0.219 |  | 0.234 |  | - |
| HPV26(n=1) |  | 0 |  | 1 |  | 0.00% |  |  |  |  |  |  |  |  |  |  |  |  |

P-values were adjusted for multiple testing using the Benjamini-Hochberg false discovery rate (FDR) at q=0.05.

★ indicates genotypes with statistically significant associations after correction

Supplementary Table S6. The immediate CIN3+ risk with different HPV genotypes in LSIL women (by Hier2.)

|  |  | **Histopathology** | | |  | **CIN3+**  **Risk** |  | **χ2** |  | **OR** |  | **95%CI** |  | **P-value** |  | **Adj.**  **Q-value** |  | **Sig.**  **(q<0.05)** |
| --- | --- | --- | --- | --- | --- | --- | --- | --- | --- | --- | --- | --- | --- | --- | --- | --- | --- | --- |
|  |  | **CIN3+** |  | **<CIN3** |  |  |  |  |  |  |  |  |  |  |  |  |  |  |
| **HPV-positive(n=3398)** |  | 317 |  | 3081 |  | 9.33% |  | 91.802 |  | 10.831 |  | 5.915-19.833 |  | <0.001 |  |  |  |  |
| **HPV-negative(n=1169)** |  | 11 |  | 1158 |  | 0.94% |  |  |  |  |  |  |  |  |  |  |  |  |
| **18 HPV Genotypes** |  |  |  |  |  |  |  |  |  |  |  |  |  |  |  |  |  |  |
| HPV16(n=859) |  | 190 |  | 669 |  | 22.12% |  | 248.715 |  | 29.898 |  | 16.16-55.314 |  | <0.001 |  | <0.001 |  | **★** |
| HPV73(n=15) |  | 2 |  | 13 |  | 13.33% |  |  |  | 16.196 |  | 3.261-80.438 |  | 0.011 |  | 0.069 |  | - |
| HPV33(n=165) |  | 19 |  | 146 |  | 11.52% |  | 68.813 |  | 13.7 |  | 6.393-29.36 |  | <0.001 |  | <0.001 |  | **★** |
| HPV45(n=28) |  | 3 |  | 25 |  | 10.71% |  |  |  | 12.633 |  | 3.319-48.085 |  | 0.004 |  | 0.036 |  | **★** |
| HPV31(n=97) |  | 8 |  | 89 |  | 8.25% |  | 27.592 |  | 9.463 |  | 3.713-24.125 |  | <0.001 |  | 0.002 |  | **★** |
| HPV18(n=304) |  | 21 |  | 283 |  | 6.91% |  | 40.418 |  | 7.812 |  | 3.723-16.389 |  | <0.001 |  | 0.002 |  | **★** |
| HPV58(n=400) |  | 26 |  | 374 |  | 6.50% |  | 39.998 |  | 7.318 |  | 3.582-14.953 |  | <0.001 |  | 0.002 |  | **★** |
| HPV52(n=557) |  | 36 |  | 521 |  | 6.46% |  | 43.43 |  | 7.274 |  | 3.674-14.403 |  | <0.001 |  | 0.002 |  | **★** |
| HPV82(n=19) |  | 1 |  | 18 |  | 5.26% |  |  |  | 5.848 |  | 0.717-47.73 |  | 0.177 |  | 0.462 |  | - |
| HPV35(n=49) |  | 2 |  | 47 |  | 4.08% |  |  |  | 4.48 |  | 0.966-20.783 |  | 0.093 |  | 0.372 |  | - |
| HPV59(n=66) |  | 2 |  | 64 |  | 3.03% |  |  |  | 3.29 |  | 0.714-15.155 |  | 0.15 |  | 0.932 |  | - |
| HPV66(n=128) |  | 2 |  | 126 |  | 1.56% |  | 0.041 |  | 1.671 |  | 0.366-7.623 |  | 0.839 |  | 0.839 |  | - |
| HPV56(n=185) |  | 2 |  | 183 |  | 1.08% |  | 0 |  | 1.151 |  | 0.253-5.233 |  | 1 |  | 1 |  | - |
| HPV53(n=195) |  | 2 |  | 193 |  | 1.03% |  | 0 |  | 1.09 |  | 0.243-4.88 |  | 1 |  | 1 |  | - |
| HPV51(n=165) |  | 1 |  | 164 |  | 0.61% |  | 0 |  | 0.642 |  | 0.082-5.004 |  | 1 |  | 1 |  | - |
| HPV68(n=94) |  | 0 |  | 94 |  | 0.00% |  |  |  |  |  |  |  |  |  |  |  |  |
| HPV39(n=71) |  | 0 |  | 71 |  | 0.00% |  |  |  |  |  |  |  |  |  |  |  |  |
| HPV26(n=1) |  | 0 |  | 1 |  | 0.00% |  |  |  |  |  |  |  |  |  |  |  |  |

P-values were adjusted for multiple testing using the Benjamini-Hochberg false discovery rate (FDR) at q=0.05.

★ indicates genotypes with statistically significant associations after correction.
